# Protein Array-Based Detection of Proteins in Kidney Tissues from Patients with Membranous Nephropathy

**DOI:** 10.1155/2017/7843584

**Published:** 2017-02-27

**Authors:** Shuqiang Wang, Yang Lu, Quan Hong, Xiaodong Geng, Xu Wang, Wei Zheng, Chengcheng Song, Chunling Liu, Meng Fan, Yue Xi, Mandi Guo, Di Wu

**Affiliations:** Department of Nephrology, PLA General Hospital, Institute of Nephrology, Beijing Key Laboratory of Kidney Disease, State Key Laboratory of Kidney Diseases, National Clinical Research Center for Kidney Diseases, Beijing 100853, China

## Abstract

Membranous nephropathy (MN) is an autoimmune inflammatory disease in which proteins related with plenty of biological processes play an important role. However, the role of these proteins in the pathogenesis of MN is still unclear. This study aimed to screen differential proteins in kidney tissue samples from MN patients by using protein arrays and determine the pathways involved in the pathogenesis of MN. This study first tested a quantitative protein array (QAH-INF-3) and two semiquantitative protein arrays (L-493 and L-507) with normal renal tissue and identified L-493 as the most appropriate assay to compare protein levels between MN tissues and normal control tissues. The L-493 array identified 66 differentially expressed proteins (DEPs) that may be associated with MN. The gene oncology (GO) and protein-protein interaction (PPI) analyses revealed several processes potentially involved in MN, including extracellular matrix disassembly and organization, cell adhesion, cell-cell signaling, cellular protein metabolic process, and immune response (*P* < 0.05). We suggest that these different pathways work together via protein signaling and result in the pathogenesis and progression of MN.

## 1. Introduction

Membranous nephropathy (MN) is an autoimmune inflammatory disease and the primary cause of nephrotic syndrome in adults, contributing to 20%–40% of end stage renal disease (ESRD) cases [1]. The characteristic pathological manifestation of MN is thickening of the glomerular basement membrane (GBM), which originates from glomerular visceral epithelial cells and endothelial cells. This thickening is due to deposition of immune complex IgG and C3 under the podocytes. However, the pathways involved in the process of GBM thickening are still unclear.

According to the previous study, autoantibodies and complements play important roles in the development and progression of MN [2]. In 2009, Beck Jr. et al. identified the anti-M-type phospholipase A2 receptor (anti-PLA2R) as an autoantibody target in approximately 70% of patients with idiopathic MN [3]. The detection of anti-PLA2R antibodies has been used clinically. The role of the membrane attack complex of the complement has been confirmed [4–6]. Proteins, which act as the executor of cell function, may take part in the whole pathogenetic process of MN. The present study concentrated on several families of cytokines such as the interleukin family, tumor necrosis factor (TNF) family, and transforming growth factor (TGF) family; nonetheless, large-scale screening and function analysis of kidney proteins are rare [7–13].

Protein microarrays are advanced and efficient technology to study proteins involved in kidney disease systematically because the arrays have the potential to screen thousands of proteins in a high-throughput manner with a smaller sample as compared with mass spectrometry (MS) and other traditional methods such as western blot and enzyme-linked immunosorbent assays (ELISAs) [14, 15]. According to the working principle, protein arrays can be classified as direct detection and indirect detection. The direct detection method could detect the proteins captured directly. The indirect detection method detects the proteins with antibody and markers such as biotin and fluorescein. In our study, we compared two different indirect methods, including the quantitative array Quantibody® Human Inflammation Array 3 (QAH-INF-3) and two semiquantitative L-series antibody arrays L-507 and L-493 with the goal of determining a suitable protein array to detect proteins in kidney tissue and perform a large-scale screen for MN-related proteins and pathways.

## 2. Materials and Methods

### 2.1. Sample Collection

Our study was approved by the institutional research ethics committee of the Chinese People's Liberation Army General Hospital (No. S2015-061-01). All procedures performed in this study involving human participants were in accordance with the ethical standards of the institutional and national research committee and with either the 1964 Declaration of Helsinki and its later amendments or the comparable ethical standards. All of the samples were obtained from the Nephrology Department of the Chinese People's Liberation Army General Hospital. A total of 15 kidney samples harvested via percutaneous renal biopsy and nephrectomy were included in the study as a discovery set: six samples from idiopathic MN patients and nine normal samples from renal cell carcinoma (RCC) patients. All of the MN patients are IMN diagnosed explicitly by renal biopsy pathology with exceptions for other diseases such as autoimmune diseases, infection, diabetes, and hypertension. There are 25, 28, and 32 glomeruli in each quality control specimen, respectively.

### 2.2. Protein Extraction

After the fresh samples were collected, the impurities and blood were washed out with PBS immediately. The process of centrifugation and removal of the supernatant was repeated for the samples until the tissue became nearly colorless. The tissue was dissociated and lysed according to the manufacturer's instructions. After the lysates were centrifuged, the protein concentrations in the supernatant were detected by using BCA methods (BCA Protein Assay Kit, Pierce, Prod #: 23227, France). The samples were diluted in sample buffer to the desired concentration in a volume of 100 *μ*L.

### 2.3. Protein Microarray Detection

We chose the three following protein arrays to compare their performance in assessing the protein levels in kidney tissue: the quantitative protein chip QAH-INF-3 (RayBiotech, Inc., Norcross, GA) and the semiquantitative protein L-series antibody array chips L-507 and L-493 (RayBiotech, Inc., Norcross, GA). QAH-INF-3 is a type of multiplexed sandwich ELISA-based quantitative array platform that enables researchers to accurately and simultaneously detect the concentration of 40 proteins by building a standard curve with a serial dilution of standard samples. L-507 and L-493 could separately detect 507 and 493 proteins based on biotin labeling and laser fluorescence scanning techniques, respectively. For detection, 12.5 *μ*g and 6.25 *μ*g samples from a single tissue were used for QAH-INF-3, and 50 *μ*g and 25 *μ*g from the same kidney sample were used for L-507 and L-493.

Protein hybridization was conducted with the QAH-INF-3, L-507, and L-493 arrays according to the respective manufacturer's instructions. The fluorescent images were scanned by an Axon GenePix (Molecular Devices, Inc., Orleans, USA), and the data were analyzed with GenePix Pro 6.0 software (Molecular Devices, Inc., Orleans, USA).

Hybridization proceeded according to the manufacture of QAH-INF-3, L-507, and L-493. The signal fluorescence images were scanned by Axon GenePix (Molecular Devices, Inc., Orleans, USA). The data was analyzed with software GenePix Pro 6.0 (Molecular Devices, Inc., Orleans, USA).

### 2.4. ELISA Verification

To verify the results, EpCAM-1, CTSD (Abcam, Inc., Cambridge, UK), and ADAMTS-4 (R&D Systems, Inc., Minnesota, USA) were selected as confirmation markers and tissue lysates of MN and N were measured by ELISA kits. The procedures were carried out following the specification of the kits.

### 2.5. Immunohistochemistry Verification

To avoid the false overexpression of transmembrane proteins caused by tissue lysates, we performed immunohistochemistry of MN and normal control with CTSD and CD46. Immunohistochemistry was carried out following the conventional methods of fixing tissues, paraffin-embedding, serial section (3 mm), antigen retrieval, blocking endogenous peroxidase activity, adding antibodies, incubation, and microscopic examination. The antibodies of CTSD and CD46 are both mouse monoclonal antibody (Santa Cruz Biotec, Inc., USA).

### 2.6. Statistical Analyses

The protein array performances among QAH-INF-3, L-507, and L-493 were assessed by the fold change of protein expression between the two concentrations of the loading samples. We defined proteins with a ratio between 1.3 and 3.0 (12.5 *μ*g versus 6.25 *μ*g group in QAH-INF-3, 50 *μ*g versus 25 *μ*g group in L-507 and L-493; the theoretical ratio was 2.0) as measurable proteins in kidney tissue and counted the number of available proteins on each chip to assess the performance of protein chips.

Student's *t*-test (*t*-test) was used to address the significance of the quantitative data. Proteins with a fold change of MN/N > 1.4 and a *P* value < 0.05 were considered to be differentially expressed proteins (DEPs). The Database for Annotation, Visualization and Integrated Discovery v6.8 Beta (DAVID v6.8 Beta, https://david-d.ncifcrf.gov) was used for gene oncology (GO) analysis. The Search Tool for the Retrieval of Interacting Genes/Proteins v10 (STRING v10, http://www.string-db.org/) was applied for protein-protein interaction analysis.

## 3. Results and Discussion

### 3.1. Results

The general clinical characteristics of MN patients and normal control subjects are shown in Table 1. The gender ratios are the same (female : male = 2 : 1), and there was no significant difference regarding age, serum creatinine (Scr), and estimate glomerular filtration rate (*P* > 0.05) while the urine protein and albumin (Alb) showed significant differences (*P* < 0.05). The sample selection was consistent with the clinical situations of MN patients and normal control subjects.

#### 3.1.1. L-493 Shows a Good Performance for Kidney Samples Compared to QAH-INF-3 and L-507

We first compared the performance of the three arrays in detecting proteins in normal kidney samples.

For QAH-INF-3, the fluorescence image, standard curves, and scatter plot are shown in Supplementary Figure 1 in Supplementary Material available online at https://doi.org/10.1155/2017/7843584. All 40 proteins could be detected in QAH-INF-3 and the exact signal intensities analysis showed that only 22.5% (9/40) of the signal intensity ratios of 12.5 *μ*g/6.25 *μ*g were between 1.3 and 2.0. Obviously, QAH-INF-9 does not meet our requirements.

For L-507, the fluorescence image and the signal values are shown as Supplementary Figure 2. The average ratio of the 50 *μ*g/25 *μ*g signal intensities was 0.99, which was far less than the theoretical ratio of 2.0. The exact signal intensities ratio of 50 *μ*g/25 *μ*g showed that 26 (5.13%) proteins were not detected, merely 61 (12.03%) were between 1.3 and 3.0, 411 (91.07%) were less than 1.3, and 9 (1.78%) were larger than 3. The majority of the ratios are biased to the lower value.

**Figure 1:**
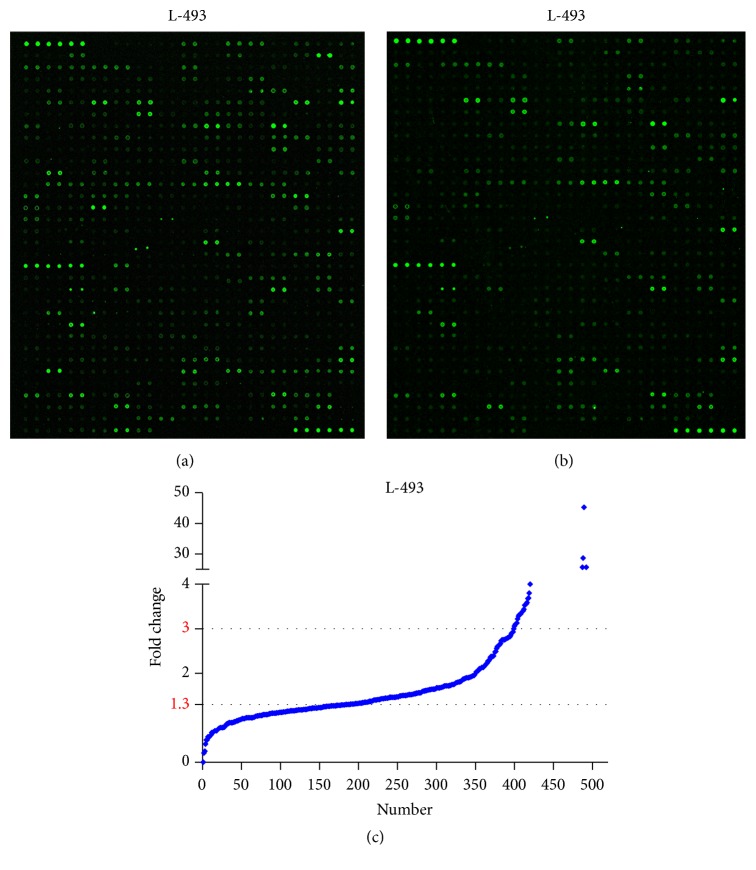
Fluorescence images and scatter plot of the semiquantitative protein array L-493 (493 proteins) in detecting proteins in kidney tissue. (a) and (b) are fluorescence images of 50 *μ*g and 25 *μ*g of the sample, respectively. (c) represents the scatter plot of L-493. There are 214 proteins with a fold change between 1.3 and 3.0.

For L-493, the fluorescence image and scatter plot are shown in Figure 1. The average ratio of the 50 *μ*g/25 *μ*g signal intensities was 2.27, which is close to the theoretical ratio of 2.0. The exact signal intensity ratios of the 50 *μ*g/25 *μ*g results showed that 4 (0.81%) proteins were not detected, 214 (43.41%) were between 1.3 and 3.0 (Figure 1(c)), 184 (37.32%) were between 0 and 1.3, and only 9 (18.46%) were larger than 3.

The primary analysis showed that L-493 could detect more available proteins (214, 43.41%) compared to QAH-INF-3 (9, 22.5%) and L-507 (61, 12.03%), suggesting that L-493 is more suitable for detecting proteins in kidney tissue. Therefore, we selected L-493 to detect DEPs in tissues from MN patients and normal controls.

#### 3.1.2. DEPs in MN Detected by L-493 Play a Crucial Role for MN Progression via Regulation of a Molecular Network

**Figure 2:**
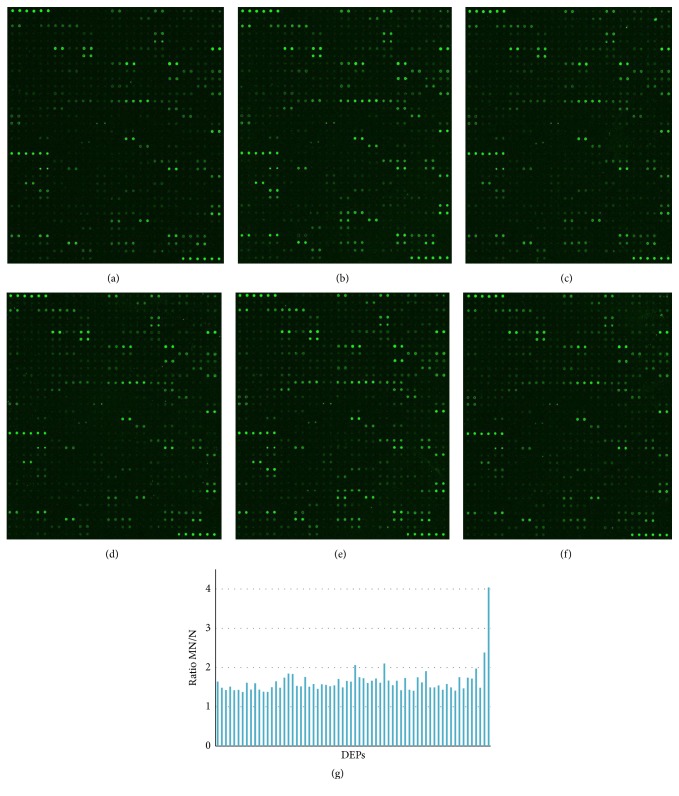
Fluorescence images and histogram of L-493 in MN versus N. (a), (b), and (c) are fluorescence images of 3 cases in normal control group; (d), (e), and (f) are 3 cases in MN. (g) is the histogram showing the ratio of MN/N in 66 upregulated different expression proteins (DEPs).

**Figure 3:**
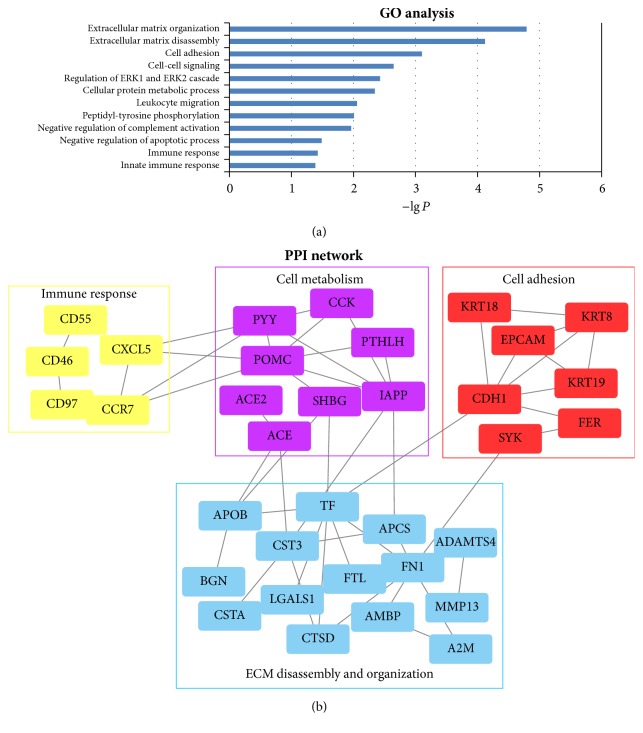
Gene oncology and predicted protein-protein interaction network of the differentially expressed proteins (DEPs). (a) shows the first 12 GO pathways (*P* < 0.05) of DEPs; the *y*-axis represents −lg⁡*P*. (b) shows the PPI network of main proteins, with different colors representing different functional zones: yellow, immune response; purple, cell metabolism; red, cell adhesion; and blue, extracellular matrix disassembly and organization.

The fluorescence images of MN and normal control were shown in Figure 2. L-493 identified 66 DEPs (fold change of MN/N > 1.4, *P* < 0.05), and they were all upregulated in MN compared to normal tissue (Figure 2(g)). The exact data was in Supplementary Table 1. GO analysis (Figure 3(a)) found several significant biological processes associated with these proteins, including extracellular matrix disassembly and organization, cell adhesion, cell-cell signaling, cellular protein metabolic process, and the immune response (*P* < 0.05).

More importantly, we constructed a molecular network based on protein-protein interactions (PPI) by using STRING (Figure 3(b)). The network and GO analysis (Table 3) showed that proteins such as EpCAM, FER, SYK, CDH1, KRT8, KRT19, and KRT18 are associated with cell adhesion; hormones such as POMC, IAPP, PTHLH, and ACE are associated with cell metabolism; CXCL5, CCR7, CD55, CD46, and CD47 are associated with immune activation; and TF, FN1, ADAMTS4, APCS, CTSD, and MMP13 are associated with extracellular matrix remodeling. What is more, this entire functional zone is interconnected by mutual proteins.

#### 3.1.3. Verification Results of ELISA and Immunohistochemistry

**Figure 4:**
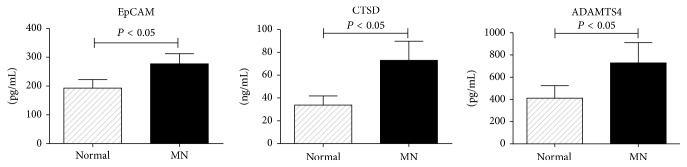
ELISA verification of EpCAM, CTSD, and ADAMTS4. The *y*-axis represents the sample concentrations. The levels of these proteins from MN tissue were higher than those in normal control tissue (*P* < 0.05).

**Figure 5:**
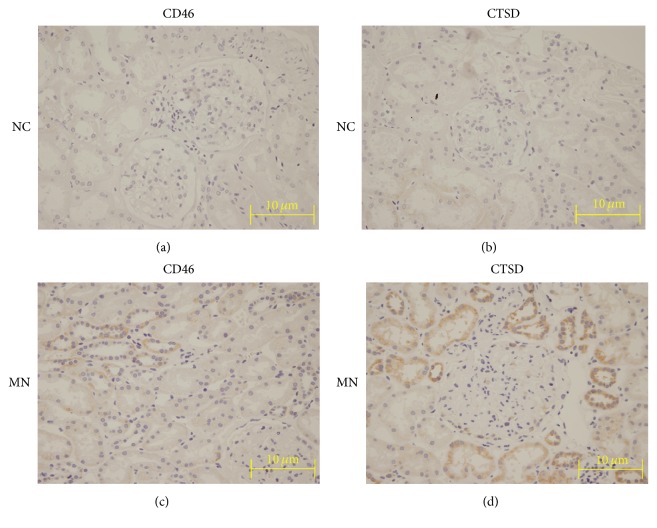
Immunohistochemistry verification of CD46 and CTSD. The images are at 400x magnification. The expressions of CD46 (a) and CTSD (b) in normal control tissues are almost negative. CD46 is partly overexpressed in tubulointerstitium of MN (c); CTSD is widely positive expressed in tubulointerstitium and slightly positive in glomerulus of MN (d).

As shown in Table 2 and Figure 4, the ratio of MN/N in EpCAM, CTSD, and ADAMTS4 is 1.44, 2.16, and 1.77 in ELISA (*P* < 0.05), similar to 1.75, 1.91, and 2.06 in L-493, respectively. And immunohistochemistry also showed the overexpression of CTSD and CD46 in MN (Figure 5). CTSD is widely overexpressed in tubulointerstitium and slightly in glomerulus (Figure 5(d)), while CD46 has a partly enhanced impression in tubulointerstitium (Figure 5(c)). These confirmed our results of protein microarray L-493.

### 3.2. Discussion

In this study, we explored the use of different protein chips for testing kidney tissues and performed a large-scale screen of proteins in kidney tissues from MN patients to elucidate the mechanism of the pathophysiological process.

We first tested three different protein chips. It was found that L-493 was most suitable for detection proteins in kidney tissue. Compared with the QAH-INF-3 and L-507 chips, we deduced that the proteins examined by L-493 were abundant in kidney tissue and that most of these proteins could be detected. Moreover, the different qualities of the antibodies in the protein chip were also important. Next, we examined the DEPs by using the L-493 chips.

We found that proteins such as CXCL5, CCR7, CD55, and CD97 were associated with the immune response. It was reported that CXCL5 and CCR7 were involved in immunocyte activation and inflammation after bacteria and virus infection [16, 17]. CD55 is the decay accelerating factor for complement; it is involved in the regulation of the complement cascade. CD97 takes part in the cell-cell signal transferring in immune activation. MN has high relevance with infection and immunity; autoantibody and proteins take the essential role in it [18]. Immune activation acts as the initiative factor in the physiopathology of MN. Borza suggested that immune response may act directly on podocytes and endothelial cells to secrete extracellular matrix and thicken the GBM [5].

GBM disassembly and reorganization are the final pathological changes of MN. We identified proteins such as MMP-13, FN1, CTSD, and ADAMTS4, which are associated with GBM disassembly and reorganization. For instance, MMP-13 is the member of the matrix metalloproteinase family that contributes to the breakdown of extracellular matrix in tissue remodeling, and previous studies have shown that this family may be associated with collagen-induced arthritis and glioblastoma [19–22]. Both arthritis and glioblastoma involve matrix disassembly in a similar manner as MN. We suggested that MMP-13 may play the same role in MN. Fibronectin 1 (FN1) may be a major component of the excessive accumulation of extracellular matrix (ECM) in the kidney glomeruli. Deltas et al. reported that FN1 is associated with thin basement membrane nephropathy (TBMN) [23]. Herbach et al. and Alvarez et al. reported increased glomerular expression of FN1 in mice and humans with diabetic nephropathy [24, 25]. Cathepsin D (CTSD) is a protein with the activity of proteolysis; it is mostly overexpressed in tubulointerstitium and the GO analysis in our research found it important in ECM remodeling. This has been supported by several previous researches about chronic kidney disease [26, 27]. We suggested that CTSD may enroll in the interstitial fibrosis after transdifferentiation of renal tubular epithelial cells induced by heavy proteinuria in MN.

Proteins such as CDH1, EpCAM, and the KRT family may be involved in cell adhesion in MN. According to previous studies, cell adhesion plays a role in glomerulonephritis, but its mechanism is still under investigation [28, 29]. Zheng et al. suggested that CDH1 contributes to the development of diabetic nephropathy (DN) [30]. Because the main pathological change during the early stages of DN is the thickening of the GBM and ECM expansion (similar to MN), we deduced that cell adhesion plays a role in MN.

Interestingly, in our results, hormones such as POMC, PTHLH, CCK, and IAPP are associated with cell metabolism in MN. We checked the GENECARDS database and found that all of these proteins are expressed in kidney. Previous studies found that PTH affects podocyte function [31], but the exact mechanism of how hormones affect GBM is unclear. We hypothesized that hormones changed the metabolism of podocytes and endothelial cells to promote the thickening of the GBM.

More importantly, we built a molecular network to elucidate the mechanism of MN. Within this network, immune activation, cell adhesion, cell-cell signal transduction, and cell metabolism could act on the ECM and promote the reorganization of the GBM. Although how these pathways interact is still unclear, we found several proteins that may serve as the link among them. For instance, spleen tyrosine kinase (SYK), as an important component of the intracellular signaling pathway for various immunoreceptors, could also regulate cell adhesion [32]. Serotransferrin (TF) is always upregulated during serious infection but may stimulate cell proliferation and matrix remodeling as well [33]. Alpha-2-macroglobulin (A2M) is a protease inhibitor and protein transporter, but GO analysis suggests its involvement in ECM organization and cell adhesion. Therefore, we believe that different pathways interact with each other and concomitantly contribute to the pathogenesis and progression of MN.

It should be mentioned that our study has some limitations. First, the protein microarray has some deficiencies, of which the most important one is that it cannot distinguish the protein location from glomeruli and tubules in kidney. Second, acquiring samples was really challenging, and high cost of the protein chip limited the sample size in the experiments and validation. In addition, we were also unable to compare multiple disease groups such as MN with IgA nephropathy, minimal change disease (MCD), and focal segmental glomerular sclerosis (FSGS). More studies are needed to address this in the future.

## 4. Conclusion

In conclusion, we used the protein array to identify differential proteins expressed in kidney tissue from MN patients, screened out proteins that may be related with MN, and reported several possible pathways that could be involved in ECM accumulation. This study is a preliminary exploration, the differential expressed proteins and pathways supply us with more targets to study MN, and we hope to continue pursuing this discovery with more thorough research.

## Supplementary Material

The supplementary material provides the proformences of QAH-INF-3 and L-507 with pictures, and the table list of 66 DEPs in MN.

## Figures and Tables

**Table 1:** Clinical characteristics of MN versus NC.

	Membranous nephropathy (mean ± SD)	Normal control (mean ± SD)	*P* value
Age	28.33 ± 8.50	35.33 ± 5.51	*P* > 0.05
Urine protein (mg/dL)	500.00 ± 100.00	16.67 ± 15.28	*P* < 0.05
Scr (umol/L)	59.90 ± 16.07	71.27 ± 15.45	*P* > 0.05
Alb (g/L)	30.30 ± 3.19	43.13 ± 2.51	*P* < 0.05
eGFR mL/(min·1.73 m^2^)	159.33 ± 23.76	119.53 ± 12.07	*P* > 0.05

**Table 2:** Results of ELISA verification.

Protein	MN	N	Fold change	*P* value
EpCAM	277.20 ± 35.32 pg/mL	192.85 ± 29.59 pg/mL	1.44	<0.05
CTSD	73.07 ± 16.75 ng/mL	33.76 ± 8.04 ng/mL	2.16	<0.05
ADAMST-4	728.50 ± 163.75 pg/mL	411.50 ± 112.96 pg/mL	1.77	<0.05

**Table 3:** GO pathways of differentially expressed proteins.

Term	*P* value	Genes
Extracellular matrix organization	1.6 × 10^−5^	FN1, ADAMTS4, CDH1, BGN, CTSD, MMP13, IBSP, A2M, CD47
Extracellular matrix disassembly	7.6 × 10^−5^	FN1, ADAMTS4, CDH1, CTSD, MMP13, A2M
Cell adhesion	7.9 × 10^−4^	FN1, CNTN2, APC, FER, EPHA3, IBSP, CD97, CD47
Cell-cell signaling	2.3 × 10^−3^	PYY, PTHLH, IAPP, CXCL5, CD97, POMC
Regulation of ERK1 and ERK2 cascade	3.7 × 10^−3^	EPHB1, FN1, SYK
Cellular protein metabolic process	4.5 × 10^−3^	BIRC5, B2M, IAPP, MMP13, ACE2, CD55, APCS, ACE, CST3, POMC
Leukocyte migration	8.8 × 10^−3^	FN1, APOB, CD244, CD47
Peptidyl-tyrosine phosphorylation	9.9 × 10^−3^	EPHB1, FER, ROR1, EPHA3
Negative regulation of complement activation	1.1 × 10^−2^	CD46, CD55
Negative regulation of apoptotic process	3.3 × 10^−2^	BIRC5, FN1, ANGPTL4, KRT18, BNIP2, ACSTD1
Immune response	3.8 × 10^−2^	TNFSF13B, DEFB1, CCR7, CXCL5, CD97
Innate immune response	4.1 × 10^−2^	B2M, DEFB1, SYK, CD244, FER, CD46, CD55, APCS
