# Supplementary material for: Protein Array-Based Detection of Proteins in Kidney Tissues from Patients with Membranous Nephropathy
Source: Biomed Res Int. 2017 Feb 27;2017:7843584. doi: 10.1155/2017/7843584 (PMC5350302; doi:10.1155/2017/7843584)
Supplement: Supplementary file 1 — The supplementary material provides the proformences of QAH-INF-3 and L-507 with pictures, and the table list of 66 DEPs in MN. [file 7843584.f1.docx]

**Protein array-based detection of cytokines in kidney tissues from patients with membranous nephropathy**

**Shuqiang Wang · Yang Lv · Quan Hong · Xiaodong Geng · Xu Wang · Wei Zheng · Chengcheng Song · Chunling Liu · Meng Fan · Yue Xi · Mandi Guo · Di Wu**

S. Wang · Y. Lv · Q. Hong · X. Geng · X. Wang · W. Zheng · C. Song · C. Liu · M. Fan · Y Xi · M. Guo · D. Wu(✉)

Department of Nephrology, PLA General Hospital, Institute of Nephrology, Beijing Key Laboratory of Kidney Disease, State Key Laboratory of Kidney Diseases, National Clinical Research Center for Kidney Diseases, Beijing, 100853, China

e-mail: wudi@301hospital.com.cn

Tel: +86-10-55499123

**Supplementary Table 1** List of 66 differentially expressed cytokines

| Full Name | UniProt ID | Official Symbol | MN/N | P-value |
| --- | --- | --- | --- | --- |
| Hydroxyacyl-CoA dehydrogenase | P40939 | HADHA | 4.04 | 0.032 |
| Parathyroid hormone-like hormone | P12272 | PTHLH | 2.38 | 0.008 |
| Squamous cell carcinoma antigen recognized by T cells | O43290 | SART1 | 2.10 | 0.050 |
| A Disintegrin-Like And Metalloprotease (Reprolysin Type) With Thrombospondin Type 1 Motif, 4 | O75173 | ADAMTS4 | 2.06 | 0.012 |
| Heat shock protein, alpha-crystallin-related, B6 | O14558 | HSPB6 | 1.97 | 0.029 |
| Cathepsin D | P07339 | CTSD | 1.91 | 0.021 |
| Ephrin receptor A3 | P29320 | EPHA3 | 1.85 | 0.039 |
| aspartate beta-hydroxylase | Q12797 | ASPH | 1.84 | 0.005 |
| CD46 molecule, complement regulatory protein | P15529 | CD46 | 1.76 | 0.018 |
| Cholecystokinin | P06307 | CCK | 1.75 | 0.041 |
| Epithelial cell adhesion molecule | P16422 | TACSTD1 | 1.75 | 0.042 |
| Cytokeratin 18 | P05783 | KRT18 | 1.75 | 0.013 |
| Adenomatous polyposis coli | P25054 | APC | 1.74 | 0.047 |
| Sclerostin | Q9BQB4 | SOST | 1.74 | 0.012 |
| Angiotensin I converting enzyme | P12821 | ACE | 1.73 | 0.028 |
| Growth/differentiation factor 2 | Q9UK05 | GDF2 | 1.73 | 0.025 |
| Biglycan | P21810 | BGN | 1.72 | 0.045 |
| Survivin | O15392 | BIRC5 | 1.72 | 0.000 |
| Peptide YY | P10082 | PYY | 1.71 | 0.004 |
| Angiopoietin-like 4 | Q9BY76 | ANGPTL4 | 1.67 | 0.012 |
| Tumor necrosis factor (ligand) superfamily, member 13b | Q9Y275 | TNFSF13B | 1.66 | 0.022 |
| Thy-1 cell surface antigen | P04216 | THY1 | 1.66 | 0.000 |
| Cadherin 1 | P12830 | CDH1 | 1.66 | 0.025 |
| Chemokine (C-C motif) receptor 7 | P32248 | CCR7 | 1.65 | 0.004 |
| EPH receptor B1 | P54762 | EPHB1 | 1.64 | 0.044 |
| CD55 molecule, decay accelerating factor for complement (Cromer blood group) | P08174 | CD55 | 1.64 | 0.017 |
| Cytokeratin 8 | P05787 | KRT8 | 1.62 | 0.009 |
| Sex hormone-binding globulin | P04278 | SHBG | 1.61 | 0.048 |
| Spleen tyrosine kinase | P43405 | SYK | 1.61 | 0.006 |
| Natural killer cell receptor 2B4 | Q9BZW8 | CD244 | 1.61 | 0.027 |
| Adenovirus E1B 19kDa interacting protein 2 | Q12982 | BNIP2 | 1.60 | 0.033 |
| Lectin, galactoside-binding, soluble, 1 | P09382 | LGALS1 | 1.58 | 0.044 |
| Cystatin C | P01034 | CST3 | 1.58 | 0.014 |
| CD47 molecule | Q08722 | CD47 | 1.57 | 0.037 |
| Apolipoprotein N | Q2KIH2 | APON | 1.56 | 0.014 |
| Claudin-3 | O15551 | CLDN3 | 1.55 | 0.022 |
| Angiotensin I converting enzyme (peptidyl-dipeptidase A) 2 | Q9BYF1 | ACE2 | 1.54 | 0.000 |
| Sex determining region Y-box 17 | Q9H6I2 | SOX17 | 1.54 | 0.009 |
| Chemokine (C-X-C motif) ligand 5 | P42830 | CXCL5 | 1.53 | 0.036 |
| Tropomyosin 1 (alpha) | P09493 | TPM1 | 1.52 | 0.034 |
| SLAM family member 8 | Q9P0V8 | SLAMF8 | 1.52 | 0.046 |
| ADAM metallopeptidase with thrombospondin type 1 motif, 19 | Q8TE59 | ADAMTS19 | 1.51 | 0.049 |
| Alpha 1 Microglobulin | Q63041 | A1M | 1.51 | 0.033 |
| Ferritin | P02792 | FTL | 1.50 | 0.023 |
| Alkaline phosphatase, placental | P05187 | ALPP | 1.49 | 0.026 |
| Apolipoprotein B | P04114 | APOB | 1.49 | 0.015 |
| Receptor tyrosine kinase-like orphan receptor 1 | Q01973 | ROR1 | 1.49 | 0.037 |
| Fibronectin | P02751 | FN1 | 1.49 | 0.047 |
| Fer (fps/fes related) tyrosine kinase | P16591 | FER | 1.48 | 0.021 |
| Pro-matrix metallopeptidase 13 | P45452 | MMP13 | 1.48 | 0.002 |
| Pleiotrophin | P21246 | PTN | 1.48 | 0.026 |
| Transferrin | P02787 | TF | 1.47 | 0.047 |
| CD97 molecule | P48960 | CD97 | 1.46 | 0.004 |
| Apelin | Q9ULZ1 | APLN | 1.44 | 0.048 |
| Endorphin Beta | P01189 | POMC | 1.44 | 0.037 |
| Contactin-2 | Q02246 | CNTN2 | 1.44 | 0.040 |
| Alpha-2-macroglobulin | P01023 | A2M | 1.43 | 0.008 |
| Proopiomelanocortin | P01189 | POMC | 1.43 | 0.020 |
| Cytokeratin 19 | P08727 | KRT19 | 1.43 | 0.049 |
| Cystatin A | P01040 | CSTA | 1.42 | 0.044 |
| Amyloid P component, serum | P02743 | APCS | 1.42 | 0.026 |
| Integrin-binding sialoprotein | P21815 | IBSP | 1.41 | 0.006 |
| Neuronal pentraxin receptor | O95502 | NPTXR | 1.41 | 0.048 |
| Ecotropic viral integration site 5-like | Q96CN4 | EVI5L | 1.38 | 0.012 |
| Defensin, beta 1 | P60022 | DEFB1 | 1.38 | 0.026 |
| Islet amyloid polypeptide | P10997 | IAPP | 1.38 | 0.021 |

Supplementary Figure 1


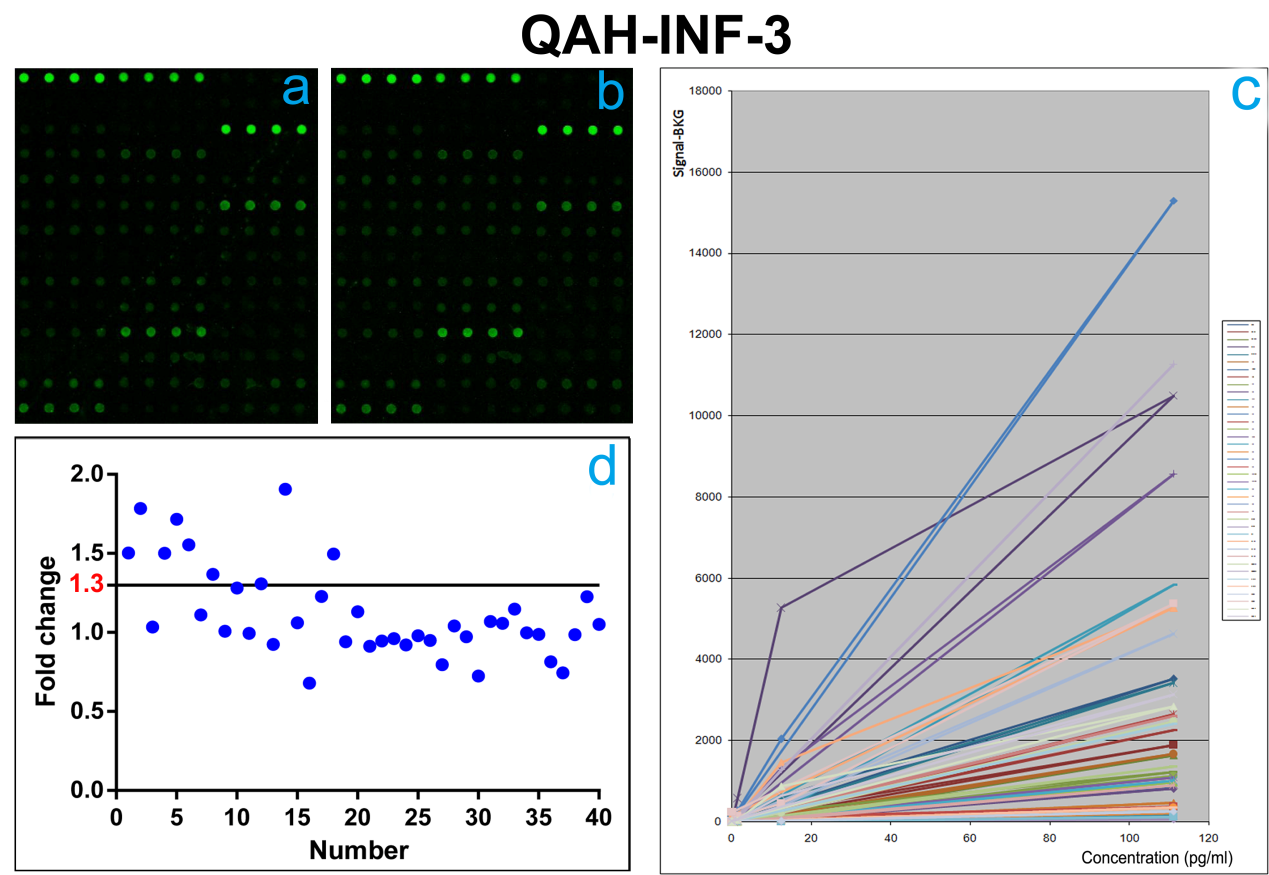


Supplementary Figure 2


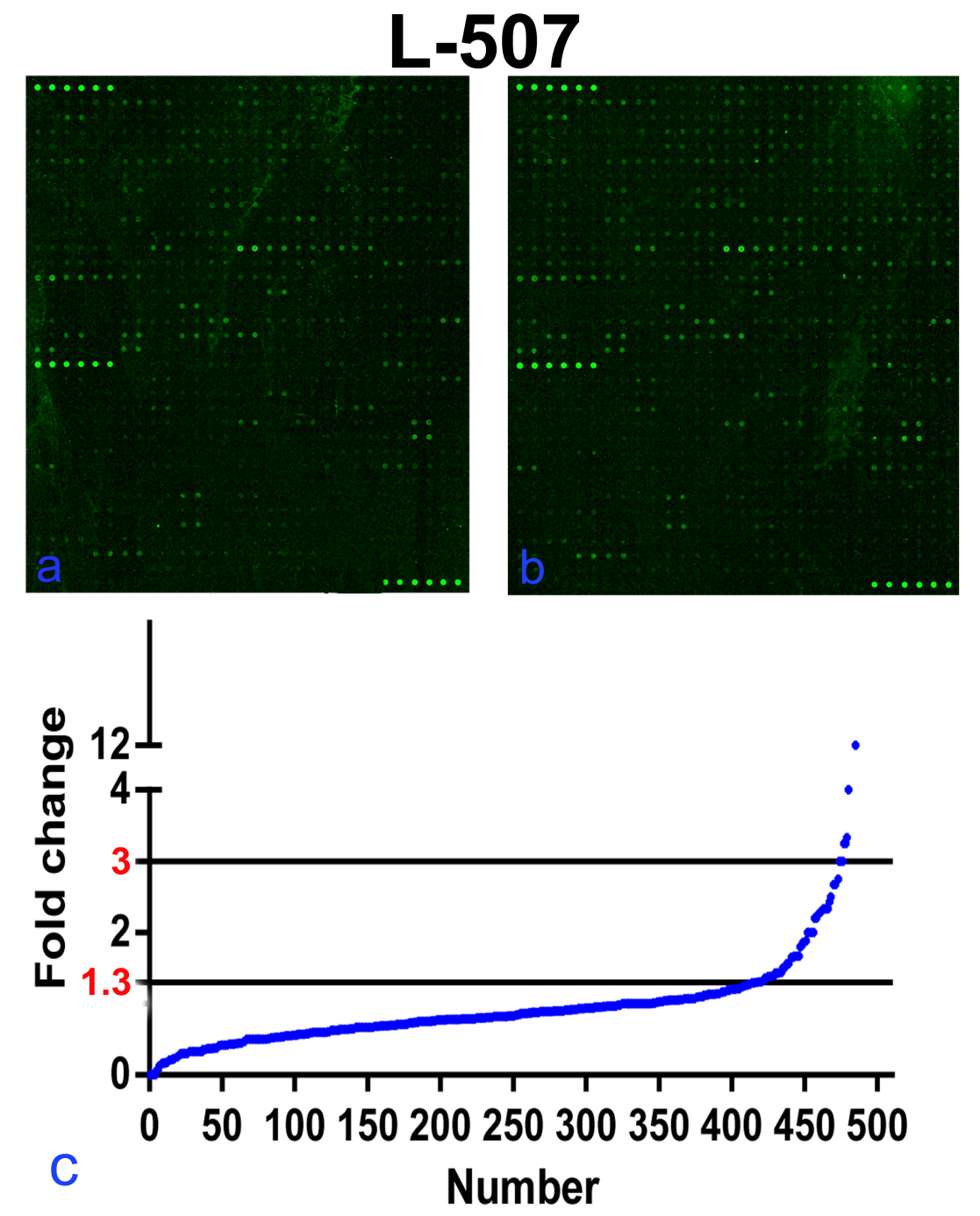


Supplementary Figure lends：

**Supplementary Fig.1** Fluorescence images, standard curves, and scatter plot of the quantitative protein array QAH-INF-3 (40 proteins) in detecting proteins in kidney tissue. **a** and **b** are fluorescence images of 12.5 µg and 6.25 µg of the sample, respectively. **c** represents the standard curves of QAH-INF-3. **d** is the scatter plot. Every point represents the 12.5 µg/6.25 µg ratio of a protein, and the points are dispersive.

**Supplementary Fig.2** Fluorescence images and scatter plot of the semiquantitative protein array L-507 (507 proteins) in detecting proteins in kidney tissue. **a** and **b** are fluorescence images of 50 µg and 25 µg of the sample, respectively. **c** represents the scatter plot of L-507.There are 61 proteins with a fold change between 1.3 and 3.0.
